# Implantation and reimplantation: epidemiology, etiology and pathogenesis over the last 30 years

**DOI:** 10.1007/s00405-024-08568-2

**Published:** 2024-03-20

**Authors:** M. C. Ketterer, K. Shiraliyev, S. Arndt, A. Aschendorff, R. Beck

**Affiliations:** 1 Department of Otorhinolaryngology–Head and Neck Surgery, Medical Center–University of Freiburg, Killianstrasse 5, 79106 Freiburg, Germany; 2 Department of Otorhinolaryngology, Vivantes, Klinikum Im Friedrichshain, Berlin, Germany

**Keywords:** Reimplantation, Implant survival, Technical upgrade, Technical failure, Medical complications, Cochlear implant

## Abstract

**Introduction:**

Due to the increasing number of cochlear implant (CI) recipients, growing indications, and the aging population, the reimplantation of CI recipients has become a focus of attention. The aim of this study is to examine the causes, timing, and postoperative speech understanding in a large cohort over the past 30 years.

**Methods:**

A retrospective data analysis was conducted on over 4000 CI recipients and 214 reimplanted children and adults from 1993 to 2020. This involved collecting and comparing data on causes, manufacturer information, and demographic data. In addition, a comparison of speech understanding in Freiburg monosyllables and numbers before and after reimplantation was carried out.

**Results:**

Children did not exhibit elevated rates of reimplantation. The overall reimplantation rate in the entire cohort was 5%. The CI overall survival rate after 10 years in the entire cohort was 95.2%. Device failure was the most common reason for reimplantation, with 48% occurring within the first 5 years after implantation. The second most common reason was medical complications, with the risk of reimplantation decreasing as more time passed since the initial implantation. There were no significant differences in Freiburg numbers and monosyllable comprehension before and after reimplantation, both in the overall cohort and in the subcohorts based on indications. Even a technical upgrade did not result in a significant improvement in speech understanding.

**Discussion:**

There was no significant difference in the frequency of reimplantation based on patient age, especially when comparing children and adults. Device failure is by far the most common indication for reimplantation, with no significant difference in implant survival between manufacturers. Patients most often choose the same manufacturer for reimplantation. The likelihood of reimplantation decreases with increasing time since the initial implantation. The indication for reimplantation should be carefully considered, as on average, no improved speech understanding is achieved, regardless of the cause, even with a technical upgrade.

## Introduction

The discussion about and the question of reimplantation are not only in focus due to increasing indications [1–4] but also because of the increasing age of our patients with cochlear implants (CIs) [5]. The indication for reimplantation is widely discussed in the literature. Minor and major criteria are distinguished, with major criteria indicating the need for surgical revision [6, 7]. Clear classifications are still not uniformly established [8].

The indications for reimplantation can be either device-related or due to a medical complication [7, 9], with device-related issues still being the main cause [10]. Device-related issues can be divided into hard versus soft failures; hard failure means that no auditory stimulation is coming from the CI anymore [11]. In cases where the device integrity check is normal, but there are persistent complaints, such as dizziness or decreased performance, it is referred to as a soft failure, and the indication for reimplantation is critically discussed [6, 9, 11]. Holcomb et al. [12] conducted a study involving ten cochlear implant (CI) patients, where they reported consistent or improved speech perception outcomes after implementing a technical upgrade. However, it is worth noting that in one of the ten patients (10%), a complete electrode array insertion was not feasible. As a result, their findings led to the conclusion that reimplantation should only be considered for individuals who are incapable of using the latest external processors due to their incompatibility with older internal devices [12]. In addition, Beck et al. [13] and Elgandy et al. [14] described that the scalar position remains the same during reimplantation, the electrode carrier should be of the same or smaller diameter than the previous one, and due to fibrosis, greater coverage cannot be achieved. In their study involving a prelingually deafened cohort (*n* = 5), Roßberg et al. [15] observed reduced cochlear coverage and a lack of improvement in speech perception, despite the utilization of advanced technology.

The aim of this work is, therefore, to compare the reasons and different indications for reimplantation to make statements about critical time windows for reimplantation and postoperative outcomes.

## Methods

A retrospective data analysis was conducted on all patients who were provided with a cochlear implant (CI) or underwent CI reimplantation or explantation at the University Hospital in Freiburg between the years 1993 and 2020. Furthermore, the analysis distinguished between primary CI placement and reimplantation. The analog and digital patient records were reviewed for collected medical history and examination findings, radiological assessments, implantation sites, operation reports, identified etiology, and general patient data (age, gender, pre-existing conditions) from 1993 to 2020. Manufacturer data (implant type, serial number, electrode carrier, side, if available, integtest) were also analyzed. To ensure audiologic comparability, Freiburg monosyllabic and numeral tests were conducted and compared in adult patients. This was done, because German speech perception tests for children vary by age and comparability.

The patients were advised independently both during the initial and the reimplantation procedures and had free choice of all implants.

We performed our statistical analyses and graphic presentations using Excel and the statistical and graphic system GNU R (ANOVA, Tukey’s Honest Significant Difference; GNU R, Version 3.0.3, Core Team, Vienna, Austria, http://www.R-project.org). The level of significance was set at 0.05, and the results were calculated descriptively. We performed the G rho-family of Harrington and Fleming (1982) test, which is a multiple comparison test, for Kaplan–Meier survival analysis.

This retrospective study was approved by the Ethics Commission of the University Hospital Freiburg (Number: 406/19, amendment: 230,282) in accordance with the Declaration of Helsinki (Washington, 2002) and is registered in the German Clinical Trials Register (www.drks.de/DRKS-Number: DRKS00019807).

## Results

### Study cohort

Between 1993 and 2020, a total of 4036 cochlear implant procedures were performed at the University Hospital Freiburg. Of these, 2279 patients were adults (56%). Children did not exhibit elevated reimplantation rates, and the overall reimplantation rate was 5%. Table 1 provides a descriptive breakdown of the implanted and reimplanted patients. During this period, a total of 214 reimplantations were conducted; 122 (5%) in adults and 92 (5%) in children. Out of these, 81 reimplantations in 76 patients were due to device malfunction, and 41 reimplantations were performed for medical reasons. Table 1 provides an overview of the percentage distribution of implantation, reimplantation, and explantation categorized by manufacturer. The recall of CI512 devices (14 in total) from CochlearTM between January and September 2011 has been excluded. The overall survival of CI devices in the entire cohort was 95.2% after 10 years and 89.4% after 20 years. On average, reimplantation occurred after 6.51 years.

**Table 1:** Study cohort and presentation of the total cohort by manufacturer with percentage distributions

	Adults (*n*)	Children (*n*)										
Implantations—total (*n*)	4036											
	2279 (56%)	1757 (44%)										
Reimplantations—total (*n*)	214											
	122 (57%)	92 (43%)										

Manufacturer (*n*)	Advanced Bionics			Cochlear™			MED EL			Oticon		
Percentage distribution	Im	Ex	Re	Im	Ex	Re	Im	Ex	Re	Im	Ex	Re
	8	10	9	74	80	81	17	9	9	1	1	1

*Im* Initial implantation; *Ex* Explantation, *Re* Reimplantation

### Reimplantation due to device failure

**Fig. 1:**
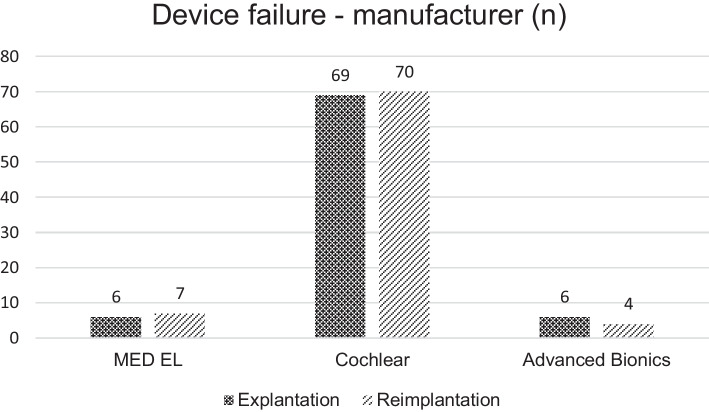
Displaying the overall distribution of manufacturer choices within the study cohort of reimplanted patients who experienced a device failure (*n* = 81). As you can observe, most patients opted for the same device for the reimplantation (right bar) compared to the initially implanted and now explanted device (left bar)

**Fig. 2:**
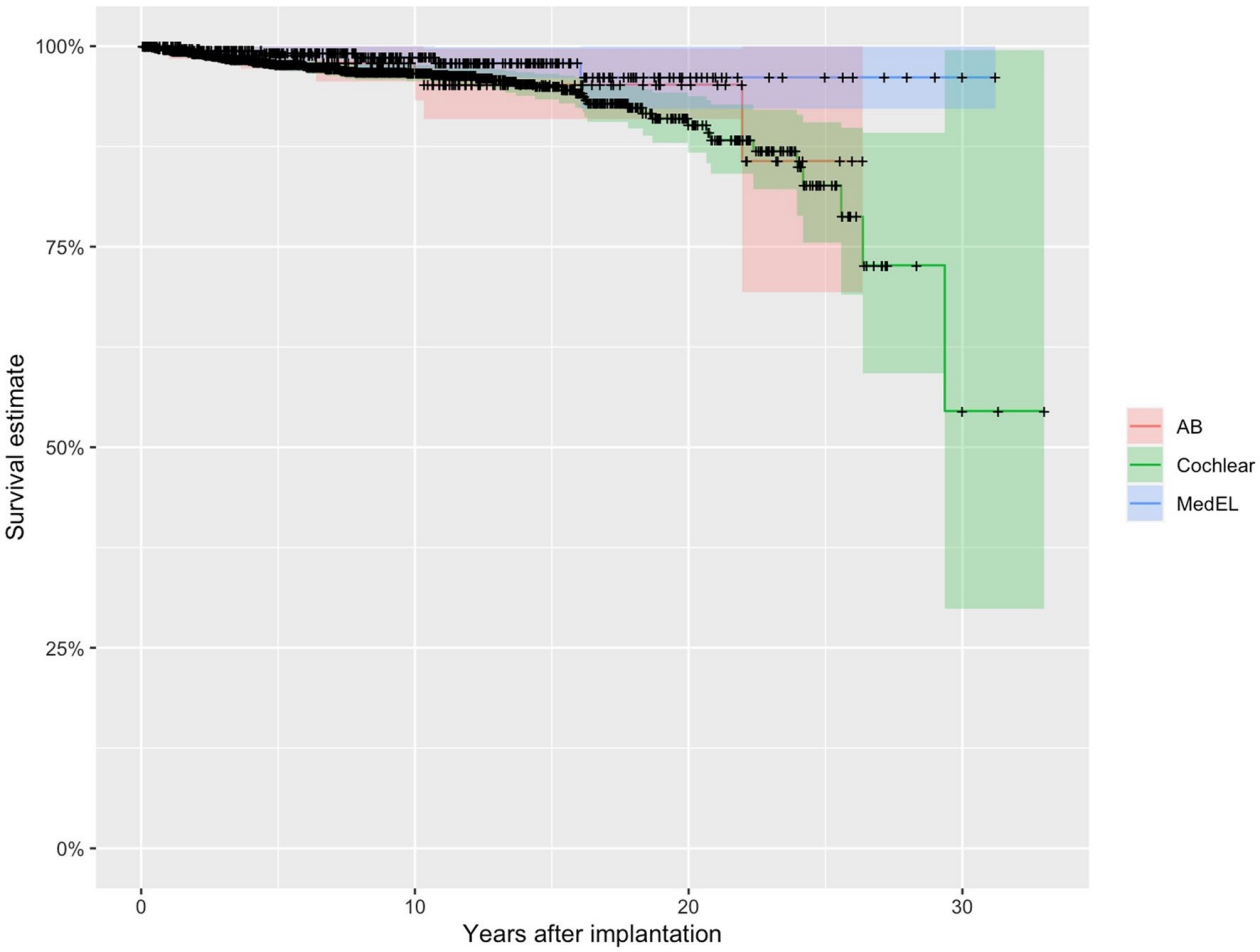
Survival estimate curves (%) in years after implantation comparing the three different manufacturers MED EL, Cochlear and Advanced Bionics (AB)

Out of the 81 reimplantations, 37 were due to hard failures (46%), and 44 (54%) were due to soft failures. Displaying the age distribution of this cohort, we could find that only 5 of the patients were in the age group over 78 years. Figure 1 illustrates the distribution of this cohort by manufacturer. 69 implants from Cochlear (85%), and 6 implants each (7%) from Advanced Bionics and MED-EL were explanted due to device failure (hard or soft). The distribution of newly reimplanted CIs was as follows: 70 (86%) Cochlear, 4 (5%) Advanced Bionics, and 7 (9%) MED-EL. Table 2 shows the distribution of the explanted device by implant type. It is evident that CI512 and CI24RECA had to be explanted most frequently due to device failure. Figure 2 presents an overall survival curve for the examined CIs by manufacturer. We could not detect a significant difference in comparison between the manufacturers (*p* = 0.05). Nevertheless, when comparing MED-EL and Cochlear™ separately, the implants from MED-EL showed significantly longer survival curves (*p* = 0.01) (Table 3).

**Table 2:** Reimplantations presented with manufacturer and implant type due to device failure

Implants	Cochlear™ CI22M: 16 CI24M: 5 CI24RCA: 3 CI24RCS: 2 CI24RECA: 11 CI24RST: 2 CI422: 2 CI512: 25 CI522: 2, Hybrid L: 1	MED EL C40 + : 3 Flex28: 1 Sonata: 2	Advanced bionics Clarion: 3 HiRes Ultra Hifocus MS: 2 HiRes90K/HiFokus: 1	Oticon /

**Table 3:** Survival rates in the reimplantation cohort after device failure, divided by manufacturer

Manufacturer	Advanced bionics (%)	Cochlear™	MED EL (%)
10-year annual survival rate	96.6	96.6	98.6
20-year annual survival rate	95.2	90.2	96.1

We could detect no significant differences when comparing all three included manufacturers. However, when comparing MED-EL and Cochlear™ separately, the implants from MED-EL showed significantly longer survival (*p* = 0.01)

Interestingly, patients almost exclusively chose the same manufacturer for reimplantation as for their initial implantation. Two patients initially implanted with Advanced Bionics switched, one to MED-EL and one to Cochlear. It is observed that 53 out of 81 reimplantations occurred within the first 10 years, with 48% occurring within the first 5 years due to device failure. Only 3 patients needed reimplantation after over 25 years of use. On average, the duration of CI use was 8.4 years until reimplantation. Table 2 presents the 10- and 20-year survival rates by manufacturer. In comparison, there were no significant differences in speech perception scores (*p* = 0.169) and monosyllabic understanding (*p* = 0.534) before and after reimplantation, despite partial technical upgrades. It can be noted that the audiograms of 42 patients can be adequately analyzed and compared. Of these, 59.5% showed an improvement compared to the preoperative speech audiogram. Nevertheless, the performance was worse in 26% (11 patients) after reimplantation. 32 out of the 42 patients (76%) received an upgrade to their speech processor, while 8 (19%) did not receive an upgrade. 2 patients could not be clearly categorized as they initially had an EAS system. Interestingly, 8 out of 11 patients (73%) who showed a deterioration in their hearing performance in the Freiburg Monosyllable Test received a technical upgrade, including an upgrade to their speech processor.

### Reimplantation due to medical complications

**Fig. 3:**
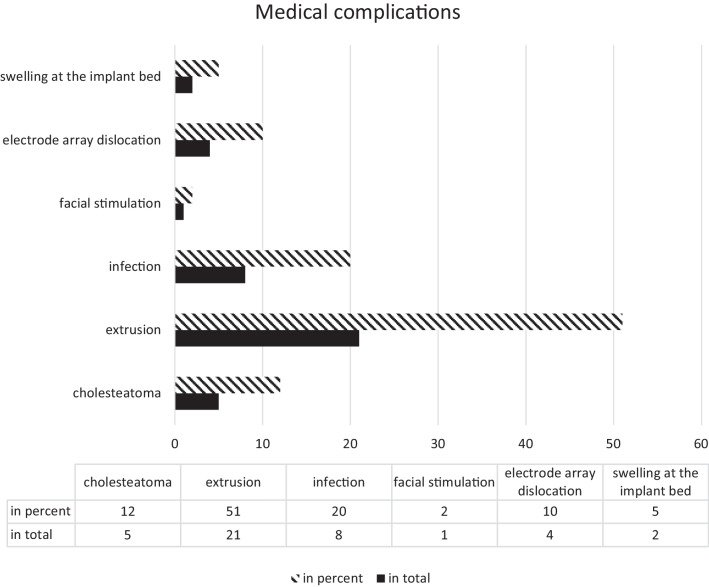
Illustration of the medical complications in total and in percent

Figure 3 presents the breakdown of reasons for medical complications leading to reimplantations and explantations in the study. The most common reason was implant extrusion. Patients with an infection of the implant were explanted initially while leaving the electrode carrier in place and were reimplanted secondarily (average: 175.6 days). Patients with cholesteatoma were also reimplanted secondarily after an uneventful second-look operation (average: 126.6 days).

The majority of reimplantations due to medical complications were performed within the first 2 years after initial implantation. The rate of reimplantations decreases with increasing time intervals after the initial implantation. Consequently, with a greater time gap from the initial implantation, the risk of needing reimplantation due to medical complications decreases. Most temporary explantations occurred within the first 2 years after implantation. Explantations within the first year following CI occurred more frequently when followed by a secondary reimplantation rather than an immediate reimplantation. In this cohort as well, there was no significant difference in speech perception scores (*p* = 0.668) and monosyllabic understanding (*p* = 0.148) before and after reimplantation.

## Discussion

### Study cohort

This study examines the probabilities, causes, and outcomes of reimplantations in a large patient cohort of 4036 initially implanted cochlear implant (CI) recipients over the past 30 years. It is one of the largest studies to investigate this question, along with Battmer et al. [16] (*n* = 3417 years). The reimplantation rate over 30 years was 5%, with no clustering in children, and it decreased over time in both the cohort with device failure and the one with medical complications. Numerous previous publications have already described that the incidence of reimplantations and revision surgeries will increase [5, 17]. The revision and reimplantation rates in some studies were slightly higher than in our study at 7.6% and 8.2% [5, 18]. In contrast, Rivas et al. [19] also described a 5% rate of necessary reimplantation surgeries. In the largest study cohort on this topic to date, there was no percentage difference in the frequency of reimplantations between children and adults. Previous work had described a greater need for reimplantations in children due to falls and trauma [5, 16, 20, 21]. Our study shows that implant durability has significantly improved, and susceptibility to fractures, among other factors, has been greatly reduced, possibly due to the replacement of ceramic casings. Rivas et al. [19] already suggested in 2008 that most reimplantations needed to occur within the first 6 years after initial implantation. This finding aligns with our study.

### Reimplantation due to device failure

Reimplantations due to device malfunction constituted the largest cohort in this study. This aligns with previous publications [18, 22, 23]. 46% were reimplanted due to hard failures, and 54% due to soft failures, although uniform classification systems are lacking, and some publications classify medical indications as soft failures [20, 24]. It is crucial to work on standardized classifications in multicenter studies for improved comparability.

Indeed, when considering MED-EL and CochlearTM separately, we found significantly longer survival curves for MED-EL. However, we did not find a significant difference when comparing all three included manufacturers. Our department began implanting CochlearTM years earlier compared to MED-EL, and consequently, initial technical issues with MED-EL implants may have potentially been resolved before we started using the first MED-EL implants. Therefore, the comparability of the included manufacturers and the survival curves must be questioned.

The CI512 and CI24RECA by CochlearTM were the most frequently explanted devices, but it is important to note the CI 512 device failure reported in 2011 and the associated recall action by Cochlear Ltd [25, 26] due to a loss of hermeticity leading to implant failure, resulting in a failure rate of 9.8% [27]. Therefore, implants produced during this period were excluded from the survival curve calculations in accordance with Reis et al. [17]. As described by Reis et al. [17], Hildrew and Molony [27] described that this raised the error rate from 2.4% to 25%, but only during that period. Therefore, in our study, this period was excluded to facilitate a valid long-term comparison. Recall actions are also known from Advanced Bionics, which had to recall its HiRes 90 k model in 2004 and 2010 due to moisture-related potential failures. Manufacturer-specific survival curves in this study show that the need for reimplantation due to device malfunction decreases with increasing time since initial implantation, consistent with previous research [10, 28–30]. These studies established an average time to reimplantation of up to 6.2 years, whereas in our study, we observed a usage duration of 8.4 years, supported by a much larger study cohort and a 30-year follow-up period. Furthermore, it can be expected that implant lifetimes will increase with technological advancements [22]. However, as described in previous work [10, 22], there was no significant difference between the included manufacturers.

Another goal of this study was to assess the postoperative outcomes, particularly in the subgroup of patients with device malfunctions. There were no significant improvements in Freiburg monosyllabic or number understanding, despite technical upgrades in 25 out of 35 evaluated and compared patients (71.4%). This is consistent with the work of Beck et al. [13], which also investigated this subset. Stable but not improved audiological results were observed despite technical upgrades. Beck et al. [13] expressed critical views on reimplantation for technical upgrade reasons, although it was shown that the reinsertion of the electrode carrier did not lead to a significantly increased risk of partial insertion, scalar dislocation, or reduced insertion angle. Lassig et al. [29] and Manrique-Huarte et al. [31] also described partial ossification and fibrosis of the cochlea, which can even lead to a loss of insertion angle and cochlear coverage upon reinsertion. The fact that even with a technical upgrade, there was no improvement in postoperative speech understanding aligns with numerous prior publications, albeit with smaller sample sizes [17, 28].

### Reimplantation due to medical complications

Reimplantation due to a medical defect was observed in 34% of the included patients, with implant extrusion being the most common reason in this subcohort. Lane et al. [32] also identified extrusion as a frequent indication for reimplantation and noted a connection between the implant bed with a bony tunnel for the electrode carrier and the risk of extrusion. Since the majority of reimplantations in this study due to medical complications occurred within the first 2 years after initial implantation, further investigations in this area are certainly warranted. It is important to distinguish extrusions from magnet displacements after MRI, which usually only require magnet revision and repositioning [33]. The likelihood of reimplantation also decreased in the cohort of medical complications with increasing time interval after initial implantation. The timing of reimplantation due to medical complications aligns with the literature [22] at 2.6 years, emphasizing the need for close postoperative follow-up and medical monitoring. In this cohort as well, there was no significant difference in postoperative speech understanding before and after reimplantation.

## Conclusions

There was no significant difference in the frequency of reimplantation concerning patient age, especially not anymore comparing children and adults. Device malfunction is by far the most common indication for reimplantation, with no significant difference in implant survival between manufacturers. Patients almost exclusively choose the same manufacturer for reimplantation. The likelihood of reimplantation decreases with increasing time since initial implantation. The decision for reimplantation should be carefully considered, since, on average, improved speech understanding is not achieved regardless of the cause, even with a technical upgrade.

## Data Availability

Data supporting this study are not publicly available. Please contact our research group.

## References

[1] Arndt S, Aschendorff A, Laszig R, Beck R, Schild C, Kroeger S, Ihorst G, Wesarg T (2011) Comparison of pseudobinaural hearing to real binaural hearing rehabilitation after cochlear implantation in patients with unilateral deafness and tinnitus. Otol Neurotol 32(1):39–47 21068690 10.1097/MAO.0b013e3181fcf271

[2] Ketterer MC, Häussler SM, Hildenbrand T, Speck I, Peus D, Rosner B, Knopke S, Graebel S, Olze H (2020) Binaural hearing rehabilitation improves speech perception, quality of life, tinnitus distress, and psychological comorbidities. Otol Neurotol 41(5):e563–e574 32068692 10.1097/MAO.0000000000002590

[3] Ketterer MC, Knopke S, Häußler SM, Hildenbrand T, Becker C, Gräbel S, Olze H (2018) Asymmetric hearing loss and the benefit of cochlear implantation regarding speech perception, tinnitus burden and psychological comorbidities: a prospective follow-up study. Eur Arch Otorhinolaryngol 275(11):2683–2693 30229458 10.1007/s00405-018-5135-9

[4] Knopke S, Häussler S, Gräbel S, Wetterauer D, Ketterer M, Fluger A, Szczepek AJ, Olze H (2019) Age-dependent psychological factors influencing the outcome of cochlear implantation in elderly patients. Otol Neurotol 40(4):e441–e453 30870379 10.1097/MAO.0000000000002179

[5] Wang JT, Wang AY, Psarros C, Da Cruz M (2014) Rates of revision and device failure in cochlear implant surgery: a 30 year-experience. Laryngoscope 124(10):2393–2399 24550135 10.1002/lary.24649

[6] Balkany TJ, Hodges AV, Buchman CA, Luxford WM, Pillsbury CH, Roland PS, Shallop JK, Backous DD, Franz D, Graham JM, Hirsch B, Luntz M, Niparko JK, Patrick J, Payne SL, Telischi FF, Tobey EA, Truy E, Staller S (2005) Cochlear implant soft failures consensus development conference statement. Otol Neurotol 26(4):815–818 16015190 10.1097/01.mao.0000178150.44505.52

[7] Venail F, Sicard M, Piron JP, Levi A, Artieres F, Uziel A, Mondain M (2008) Reliability and complications of 500 consecutive cochlear implantations. Arch Otolaryngol Head Neck Surg 134(12):1276–1281 19075122 10.1001/archoto.2008.504

[8] Jiang Y, Gu P, Li B, Gao X, Sun B, Song Y, Wang G, Yuan Y, Wang C, Liu M, Han D, Dai P (2017) Analysis and management of complications in a cohort of 1065 minimally invasive cochlear implantations. Otol Neurotol 38(3):347–351 28192378 10.1097/MAO.0000000000001302 PMC5305286

[9] Battmer RD, Backous DD, Balkany TJ, Briggs RJ, Gantz BJ, van Hasselt A, Kim CS, Kubo T, Lenarz T, Pillsbury HC 3rd, O’Donoghue GM, International Consensus Group for Cochlear Implant Reliability Reporting (2010) International classification of reliability for implanted cochlear implant receiver stimulators. Otol Neurotol 31(8):1190–1193 20864879 10.1097/MAO.0b013e3181d2798e PMC4886654

[10] Kim SY, Kim MB, Chung WH, Cho YS, Hong SH, Moon IJ (2020) Evaluating reasons for revision surgery and device failure rates in patients who underwent cochlear implantation surgery. JAMA Otolaryngol Head Neck Surg 146(5):414–420 32134441 10.1001/jamaoto.2020.0030 PMC7059107

[11] Lenarz T (2018) Cochlear implant—state of the art. GMS Curr Top Otorhinolaryngol Head Neck Surg. 16:Doc04 29503669 10.3205/cto000143 PMC5818683

[12] Holcomb MA, Burton JA, Dornhoffer JR, Camposeo EL, Meyer TA, McRackan TR (2019) When to replace legacy cochlear implants for technological upgrades: indications and outcomes. Laryngoscope 129(3):748–753 30484865 10.1002/lary.27528 PMC6379146

[13] Beck R, Shiraliyev K, Arndt S, Rauch AK, Aschendorff A, Hassepass F, Ketterer MC (2022) Scalar position, dislocation analysis and outcome in CI reimplantation due to device failure. Eur Arch Otorhinolaryngol 279(10):4853–4859 35226182 10.1007/s00405-022-07315-9 PMC9474456

[14] Elgandy MS, Mobashir MK, El-Sheikh E, Hansen M, Tyler R, Dunn C, Gantz B (2018) Revision cochlear implant surgery. Int Tinnitus J 22(2):188–197 10.5935/0946-5448.20180031

[15] Roßberg W, Timm M, Matin F, Zanoni A, Krüger C, Giourgas A, Bültmann E, Lenarz T, Kral A, Lesinski-Schiedat A (2021) First results of electrode reimplantation and its hypothetical dependence from artificial brain maturation. Eur Arch Otorhinolaryngol 278(4):951–958 32562027 10.1007/s00405-020-06125-1 PMC7954748

[16] Battmer R-D, Linz B, Lenarz T (2009) A review of device failure in more than 23 years of clinical experience of a cochlear implant program with more than 3400 implantees. Otol Neurotol 30(4):455–463 19373120 10.1097/MAO.0b013e31819e6206

[17] Reis M, Boisvert I, Looi V, da Cruz M (2017) Speech recognition outcomes after cochlear reimplantation surgery. Trends Hear 2017(21):2331216517706398 10.1177/2331216517706398 PMC5536375 28752810

[18] Masterson L, Kumar S, Kong JHK, Briggs J, Donnelly N, Axon PR, Gray RF (2012) Cochlear implant failures: lessons learned from a UK centre. J Laryngol Otol 126(1):15–21 22032544 10.1017/S0022215111002829

[19] Rivas A, Marlowe AL, Chinnici JE, Niparko JK, Francis HW (2008) Revision cochlear implantation surgery in adults: indications and results. Otol Neurotol 29(5):639–648 18665030 10.1097/MAO.0b013e31817e5d31

[20] Sterkers F, Merklen F, Piron JP, Vieu A, Venail F, Uziel A, Mondain M (2015) Outcomes after cochlear reimplantation in children. Int J Pediatr Otorhinolaryngol 79(6):840–843 25843784 10.1016/j.ijporl.2015.03.015

[21] Zeitler DM, Budenz CL, Roland JT Jr (2009) Revision cochlear implantation. Curr Opin Otolaryngol Head Neck Surg 17(5):334–338 19502980 10.1097/MOO.0b013e32832dd6ac

[22] Layfield E, Hwa TP, Naples J, Maina I, Brant JA, Eliades SJ, Bigelow DC, Ruckenstein MJ (2021) Failure and revision surgery after cochlear implantation in the adult population: a 10-year single-institution retrospective and systematic review of the literature. Otol Neurotol 42(3):408–413 33351564 10.1097/MAO.0000000000002940

[23] Wijaya C, Simões-Franklin C, Glynn F, Walshe P, Reilly R, Viani L (2019) Revision cochlear implantation: the Irish experience. Cochlear Implants Int 20(6):281–287 31369357 10.1080/14670100.2019.1647372

[24] Chung D, Kim AH, Parisier S, Linstrom C, Alexiades G, Hoffman R, Kohan D (2010) Revision cochlear implant surgery in patients with suspected soft failures. Otol Neurotol 31(8):1194–1198 20729777 10.1097/MAO.0b013e3181f0c631

[25] CochlearTM NucleusVR Reliability Report—Volume 10 (2012) http://professionals.cochlearamericas.com/cochlear-products/nucleus-cochlear-implants/reliability. Accessed 16 Dec 2012

[26] Roberts C. Update on NucleusVR CI500 series implant recall. (2011) http://www.sacig.org.za/wp-content/uploads/2013/08/Update-on-Nucleus-CI500-series-implant-recall.pdf. Accessed 18 Mar 2024

[27] Hildrew DM, Molony TB (2013) Nucleus N5 CI500 series implant recall: hard failure rate at a major cochlear implantation center. Laryngoscope 123(11):2829–2833 23674156 10.1002/lary.24149

[28] Côté M, Ferron P, Bergeron F, Bussières R (2007) Cochlear reimplantation: causes of failure, outcomes, and audiologic performance. Laryngoscope 117:1225–1235 17603321 10.1097/MLG.0b013e31805c9a06

[29] Lassig A-AD, Zwolan TA, Telian SA (2005) Cochlear implant failures and revision. Otol Neurotol 26(4):624–634 16015158 10.1097/01.mao.0000178123.35988.96

[30] Marlowe AL, Chinnici JE, Rivas A, Niparko JK, Francis HW (2010) Revision cochlear implant surgery in children: the Johns Hopkins experience. Otol Neurotol 31(1):74–82 19887981 10.1097/MAO.0b013e3181c29fad

[31] Manrique-Huarte R, Huarte A, Manrique MJ (2016) Surgical findings and auditory performance after cochlear implant revision surgery. Eur Arch Otorhinolaryngol 273(3):621–629 25814389 10.1007/s00405-015-3610-0

[32] Lane C, Zimmerman K, Agrawal S, Parnes L (2020) Cochlear implant failures and reimplantation: a 30-year analysis and literature review. Laryngoscope 130(3):782–789 31112331 10.1002/lary.28071

[33] Hassepass F, Stabenau V, Arndt S, Beck R, Bulla S, Grauvogel T, Aschendorff A (2014) Magnet dislocation: an increasing and serious complication following MRI in patients with cochlear implants. Rofo 186(7):680–685 24497090 10.1055/s-0033-1356238

